# Editorial: The pathogenesis and treatment of *Helicobacter pylori*-induced diseases

**DOI:** 10.3389/fcimb.2023.1219503

**Published:** 2023-07-03

**Authors:** Yifei Xu, Anna K. Walduck, Huafeng Pan

**Affiliations:** ^1^ Department of Gastroenterology, Shenzhen Traditional Chinese Medicine Hospital, Shenzhen, China; ^2^ Rural Health Research Institute, Charles Sturt University, Orange, NSW, Australia; ^3^ Science and Technology Innovation Center, Guangzhou University of Chinese Medicine, Guangzhou, China

**Keywords:** *Helicobacter pylori*, spasmolytic polypeptide expressing metaplasia, natural product (NP), pathogenesis, treatment

*Helicobacter pylori* (*H. pylori*) is a Gram-negative, spiral-shaped bacterium colonizing the stomach of more than half of the global population (Ansari and Yamaoka, 2022). The bacterium was discovered as a cause of chronic gastritis by Dr. Barry Marshall and Dr. Robin Warren in 1982 and became a focus of intense research in the following decades. It has been identified as the major infectious cause of atrophic gastritis, spasmolytic polypeptide-expressing metaplasia (SPEM), intestinal metaplasia (IM), atypical hyperplasia, and gastric cancer (Malfertheiner et al., 2023). Empirical triple (proton pump inhibitor + two antibiotics) and quadruple therapy (proton pump inhibitor + bismuth + two antibiotics), and even novel high-dose dual therapy (proton pump inhibitor + high dose amoxicillin), have been used to clear *H. pylori* in the clinic, but the increasing rate of drug resistance is bringing great challenges to effective bactericidal treatment worldwide (O’Connor et al., 2017; Liou et al., 2023). Gene mutation, efflux pump, coccoid and biofilm formation, and intracellular survival contribute to the known mechanisms of the bacteria against antibiotics (Kuo et al., 2017; Tshibangu-Kabamba and Yamaoka, 2021). Alternative therapies ranging from nanoparticles to phage therapy are in development, but while some plant extracts have shown promising adjuvant effects, many remain in the preclinical stage (Sousa et al., 2022).

Meanwhile, the phenomenon of heteroresistance (the presence of different drug-resistant or sensitive strains in different regions of the same stomach) of *H. pylori* may contribute to treatment failure, and current treatment recommendations do not address this issue (Mascellino et al., 2017; Rizvanov et al., 2019). A recent systematic review concluded that the prevalence of heteroresistance to the most frequently used drugs of metronidazole and clarithromycin was 7 and 14%, respectively, in *H. pylori*-positive samples (Kouhsari et al., 2022). Furthermore, the recent paradigm shift from empirical therapy to sensitivity-guided treatment recommendations (Graham and Moss, 2022) underlines that a single biopsy cannot be considered sufficient for determination of antimicrobial susceptibility (Mascellino et al., 2017). Increased sensitivity testing will lead to additional data on susceptibility patterns and a likely extension of our understanding of heteroresistance.

Other *H. pylori*-induced conditions such as intestinal metaplasia and atypical hyperplasia are difficult to reverse after late eradication treatment (Sung et al., 2020; Imai et al., 2021; Shah et al., 2021). The pressing need for new therapeutic strategies and drugs means that investigating the mechanisms by which *H. pylori* infection leads to pre-cancer and cancer is of high importance.

The infection process of *H. pylori* is complex. *H. pylori* survives acidic conditions in the stomach by producing urease, which hydrolyzes the urea and produces ammonia, neutralizing conditions in the vicinity of the bacterium (Li et al., 2019). Meanwhile, the flagella provide strong motility for *H. pylori* to penetrate the gastric mucus; this process is further enhanced by the urease enzyme, which reduces mucin viscosity (Celli et al., 2009; Sharndama and Mba, 2022). Colonization is mediated by adhesin proteins including the blood group antigen-binding adhesin (BabA) and sialic acid-binding adhesin (SabA), which bind to glycan receptors in the membrane of epithelial cell and prevent the elimination of gastric juice secretion (Bonsor and Sundberg, 2019). The type IV secretion system encoded by the Cag PAI gene island of *H. pylori* translocates the cytotoxin-associated gene A (CagA) and peptidoglycan to the host cell, triggering a cascade of signaling events including tight junction and cytoskeletal rearrangements (Sgouras et al., 2016). The other major virulence factor, vacuolating cytotoxin A (VacA), causes cell damage and is associated with severe disease, severe damage, and inhibition of T-cell responses (Takahashi-Kanemitsu et al., 2020). These extracellular bacteria respond to quorum-sensing molecules such as AI-2 to become motile spiral-shaped bacteria or resting-state coccoid forms within a biofilm (Sweeney et al., 2019). In addition, some bacteria invade the host cell and survive inside the autophagosomes, thus causing intracellular damage. Some clinical *H. pylori* strains have been reported to have strong cell invasive ability (Xu et al., 2021). In addition to killing or inhibiting the bacteria directly, the blocking of these infection processes presents a potential strategy for the treatment of *H. pylori* infection.

This Research Topic collects four articles, two of which are focused on the role of the Cag PAI system, one of which reveals the role of vitamin D3 in the inhibition of *H. pylori*, and another of which comprehensively reviews studies related to SPEM from 2002 to 2022.

Ray et al. reported on studies that demonstrate that the oxidation of curcumin is required for the growth inhibition of *H. pylori*. Bacteria such as *Escherichia coli* and *Citrobacter rodentium* that possess the *curA* gene, which promotes the reductive metabolism of curcumin, are resistant to the anti-bacterial effects of curcumin. *H. pylori* and the curA deletion mutants of *E. coli* were shown to be susceptible. Furthermore, curcumin inhibited the translocation and phosphorylation of CagA, reduced the phosphorylation of c-Src in a mouse organoid model, and decreased the expression of CXCL8 in AGS cells. Finally, *H. pylori* re-isolated from curcumin-fed mice in a phospholipid formulation appeared to be less virulent. The work provides mechanistic evidence that encourages the testing of curcumin as a dietary approach to inhibit the virulence of CagA. Although it is unlikely that the bacteria could be directly eliminated using curcumin solely, the long-lasting effects on the virulence of *H. pylori* has promising implications.

Zhou et al. used vitamin D receptor knock-down (VDR-KD) mice to explore the roles of vitamin D3 and vitamin D receptor in anti-*H. pylori* infection. Previous reports showed that vitamin D3 upregulated protein 1 deficiency promotes the development of *H. pylori*-induced gastric carcinogenesis, and *H. pylori* infection induces increased expression of the vitamin D receptor (Kwon et al., 2012; Guo et al., 2014). This study reveals the anti-*H. pylori* mechanism of vitamin D3 through enhancing the expression of vitamin receptor (VDR) and cathelicidin antimicrobial peptide (CAMP). Taken together, vitamin D3 could be used in the clinical management of *H. pylori* eradication and the possible prevention of *H. pylori*-induced tumorigenesis.

Cisplatin is one of the most powerful chemotherapeutic drugs used in clinical therapeutics, especially for the treatment of solid tumors including ovarian, testicular, and bladder cancer. Off-label uses for cisplatin include the treatment of gastric cancer and esophageal cancer. Lettl et al. employed a Cag type IV secretion reporter to screen the high-efficiency inhibitor targeting this system. Cisplatin and other platinum complexes were demonstrated to have DNA binding-independent inhibitory effects against different *H. pylori* processes including adherence and type IV secretion. This excellent study provides a new strategy to search for high-performance chemical compounds against *H. pylori* by using structural and chemical biology methods.

Spasmolytic polypeptide-expressing metaplasia (SPEM) cells are characterized by mucous cell-specific proteins such as spasmolytic polypeptide (TFF2) co-expressed with the chief cell characteristic in the basal region of the gland. Although the origin of these is still controversial, SPEM has been considered a precancerous lesion in recent years (Bockerstett et al., 2020). Liu et al. summarized publications about SPEM from between 2002 and 2022 using bibliometric analysis methods. The results showed that SPEM cell lineage differentiation, interaction with *H. pylori*, disturbances of the mucosal microenvironment, biomarkers, clinical diagnosis, and the outcomes of SPEM, as well as the development of proliferative SPEM animal models, were the major focus of research. Some rapid SPEM animal models have been established, but there is little doubt that *H. pylori* is the major pathogen causing SPEM in the clinic, indicating that concentration on *H. pylori*-induced SPEM rather than chemical triggers would promote a deeper understanding of the role of SPEM in clinical practice.

We hope this Research Topic provides valuable insight into the pathogenesis of *H. pylori* and the development of a new therapeutic strategy. In addition to focusing on the development of novel bacteriostatic and bactericidal compounds, targeting the infection and pathogenic process of *H. pylori* could also be an effective strategy for managing this chronic infection. The organic lesion induced by *H. pylori* is still a refractory disease in clinical settings, and the inhibition of SPEM malignant progression could be a therapeutic target in the prevention of gastric cancer.

## Author contributions

All authors listed have made a substantial, direct, and intellectual contribution to the work and approved it for publication.
